# Deficiency of Microglial Autophagy Increases the Density of Oligodendrocytes and Susceptibility to Severe Forms of Seizures

**DOI:** 10.1523/ENEURO.0183-20.2021

**Published:** 2021-02-02

**Authors:** Mahabub Maraj Alam, Xiao-Feng Zhao, Yuan Liao, Ramkumar Mathur, Sarah E. McCallum, Joseph E. Mazurkiewicz, Matthew A. Adamo, Paul Feustel, Sophie Belin, Yannick Poitelon, Xinjun Cindy Zhu, Yunfei Huang

**Affiliations:** 1 Department of Neuroscience and Experimental Therapeutics, Albany Medical College, Albany, NY 12208; 2 Department of Molecular and Cellular Physiology, Albany Medical College, Albany, NY 12208; 3 Department of Neurosurgery, Albany Medical College, Albany, NY 12208; 4 Department of Medicine, Albany Medical College, Albany, NY 12208

**Keywords:** autophagy, epilepsy, microglia, mTOR, myelination, seizure

## Abstract

Excessive activation of mTOR in microglia impairs CNS homeostasis and causes severe epilepsy. Autophagy constitutes an important part of mTOR signaling. The contribution of microglial autophagy to CNS homeostasis and epilepsy remains to be determined. Here, we report that ATG7KO mice deficient for autophagy in microglia display a marked increase of myelination markers, a higher density of mature oligodendrocytes (ODCs), and altered lengths of the nodes of Ranvier. Moreover, we found that deficiency of microglial autophagy (ATG7KO) leads to increased seizure susceptibility in three seizure models (pilocarpine, kainic acid, and amygdala kindling). We demonstrated that ATG7KO mice develop severe generalized seizures and display nearly 100% mortality to convulsions induced by pilocarpine and kainic acid. In the amygdala kindling model, we observed significant facilitation of contralateral propagation of seizures, a process underlying the development of generalized seizures. Taken together, our results reveal impaired microglial autophagy as a novel mechanism underlying altered homeostasis of ODCs and increased susceptibility to severe and fatal generalized seizures.

## Significance Statement

Microglia play a critical role in CNS homeostasis, predominantly by impinging on neurons and astrocytes. A role for microglia in the development of oligodendrocytes (ODCs) has begun to be recognized, but the mechanism remains largely unknown. The present study uncovered a novel role of microglial autophagy in ODC homeostasis. Importantly, we found that deficiency of microglial autophagy causes severe forms of generalized seizures and high mortality, pointing to a strong association of altered ODC homeostasis and epilepsy. Our findings have implications in understanding severe forms of epilepsy.

## Introduction

Macroautophagy/autophagy is a catabolic process that regulates cellular homeostasis via quality control of cellular proteins and organelles; it is also involved in the removal of invading pathogens and phagocytotic cellular debris (Levine and Klionsky, 2004; Rubinsztein et al., 2005). Autophagy is part of the mTOR signaling network. Autophagy activity is downregulated when mTOR is excessively activated under conditions such as the presence of growth factors, increased nutrient bioavailability, stress, and many others (Levine and Klionsky, 2004).

In the CNS, neuronal autophagy regulates homeostasis of axons and is implicated in neurodegeneration (Yamamoto and Yue, 2014). Autophagy activity is suppressed in human epilepsy conditions such as tuberous sclerosis complex (TSC), in which mTOR signaling is excessively activated (McMahon et al., 2012; Di Nardo et al., 2014; Ebrahimi-Fakhari et al., 2016). Selective deficiency of autophagy activity in neurons leads to spontaneous seizures in mouse models, suggesting that neuronal autophagy constitutes part of the mTOR signaling network in the CNS and plays a critical role in maintaining neuronal excitability (McMahon et al., 2012).

The role of microglia and the microglial mTOR signaling network in epilepsy has recently garnered significant attention (Nonoda et al., 2009; Sierra et al., 2010; Abraham et al., 2012; Sosunov et al., 2012; Brewster et al., 2013; Eyo et al., 2014; Nguyen et al., 2015; Abiega et al., 2016; van Vliet et al., 2016; Wyatt-Johnson et al., 2017; Schartz et al., 2018; Zhao et al., 2018; Mo et al., 2019). Microglial mTOR activity is elevated in human epilepsy and animal models (Sosunov et al., 2012; Liu et al., 2014; Zhao et al., 2018). Excessive activation of mTOR in TSC1-deficient mice leads to hyperproliferation of microglia and astrocytes, increased phagocytotic activity, and severe spontaneous seizures (Zhao et al., 2018). In addition to their roles in regulating synaptic pruning and engulfing dead neurons and cellular debris (Nimmerjahn et al., 2005; Paolicelli et al., 2011; Schafer et al., 2012; Abiega et al., 2016; Wyatt-Johnson and Brewster, 2020), microglia also interact with other non-neuronal cells. For example, loss of TSC1 in microglia led to astrocyte proliferation (Zhao et al., 2018). In other recent studies, microglia have been reported to regulate oligodendrocyte (ODC) proliferation and myelination of neuronal axons (Miron, 2017; Wlodarczyk et al., 2017; Lloyd and Miron, 2019). However, the exact mechanism remains to be elucidated.

In the present study, we employed a microglial specific Cre line to delete a key autophagy gene, *Atg7*, from mouse microglia (Zhao et al., 2018, 2019) and characterized the impact of microglial autophagy deficiency on CNS homeostasis and seizure susceptibility.

## Material and Methods

### Animals

Animals were housed in the pathogen-free section of the Animal Research Facility. The animal facility is temperature and humidity controlled. The animals were kept in a 12/12 h light/dark cycle period (lights on at 7 A.M.) and had *ad libitum* access to food and water. The experiments were performed according to the guidelines set by the Institutional Animal Care and Use Committee as well as the National Institutes of Health’s *Guide for the Care and Use of Laboratory Animals*. Every effort was taken to reduce animal suffering and usage. Atg7^f/f^ mice were donated by Masaaki Komatsu (McMahon et al., 2012). Cx3cr1-cre mice were acquired from the Mutant Mouse Resource and Research Center (Tg(Cx3cr1-Cre)MW126Gsat/Mmucd; stock #036395-UCD). These Cx3cr1-cre mice were shown to have a microglia specificity >99% in a recent report (Zhao et al., 2019). ATG7^Cx3cr1-creCKO^ (ATG7KO) were generated by crossing ATG7 floxed mice with Cx3cr1-cre mice. Cre-negative littermates were used as controls. Both male and female mice 8–10 weeks old were used for the experiments.

### Brain tissue collection for Western blotting, RNA, and corpus callosum isolation

Animals were injected with pentobarbital (100 mg/kg, i.p.) for anesthesia, and were transcardially perfused with 30 ml of 1× PBS. Following perfusion, brains were removed from the skull and cortical and hippocampal tissues were dissected on ice-cold 1× PBS for RNA analysis. For Western blot analysis, forebrain tissue samples were collected and frozen in dry ice immediately for later use. For isolation of corpus callosum, whole-brain samples were harvested and dissected with the aid of a dissecting microscope to isolate corpus callosum. During the isolation process, brains were kept in ice-cold 1× PBS. Following isolation, corpus callosum tissue samples were immediately frozen in dry ice until later use.

### Western blot analysis

We added 300 μl of RIPA lysis buffer (150 mm sodium chloride, 1% NP-40, 0.5% sodium deoxycholate, 0.1% SDS, and 50 mm Tris-HCl; pH 8.0) per ∼5 mg of tissue sample. Tissue was then homogenized using an electronic homogenizer. Following homogenization, constant agitation was maintained for 30 min at 4°C. Following agitation, samples were centrifuged for 20 min at 10,000 × *g* at 4°C. Supernatant containing protein was pipetted out and the pellet was discarded. Protein concentration was determined by using a Pierce BCA Protein Assay kit (according to the manufacturer’s instructions) and a SmartSpec Plus Spectrophotometer. Following protein concentration measurement, an equal volume of 2× LDS (lysis buffer) was added to the tubes containing protein. Lysed samples were then incubated in a 95°C heat block for 5 min; 12 μg of protein sample were loaded into each well of a 12% Bis-Tris gel and separated at a constant voltage of 80 V. Gels containing protein were transferred to nitrocellulose membranes in cold transfer buffer for 2 h at 4°C. Constant current of 0.4 A was applied. Following transfer, membranes were cut according to desired sizes and blocked using 5% non-fat milk in TBST (25 mm Tris-HCl, pH 7.4, 1.5 m NaCl, and 0.05% Tween 20) for 2 h at room temperature. Membranes were washed once with TBST following blocking. Primary antibodies were diluted in TBST and the membranes were incubated with primary antibody (Seen in Tables 1 and 2) overnight with mild shaking. The following morning, membranes were washed three to four times with TBST again with mild shaking, 15 min per wash. HRP-conjugated secondary antibody was diluted in 5% non-fat milk in TBST and the membranes were incubated for 2 h at room temperature. Following secondary antibody incubation, the membranes were washed three to four times as described above, and then developed using a Chemiluminescent Substrate kit (ThermoScientific, 34580). Sample size was six (three M, three F) for both control and mutant mice. Signal intensities were analyzed with ImageJ software.

### RNA isolation, RT-PCR, real-time PCR

RNA was isolated from dissected cortical and hippocampal tissues, as well as from purified microglia. RNA was isolated by using TRIzol reagent according to the manufacturer’s instructions. Tissue samples were homogenized in TRIzol before proceeding with the manufacturer’s instructions. RNA pellets were re-suspended in 50 μl of RNase-free water and incubated at 55°C for 5 min. RNA concentrations were measured by using a SmartSpec Plus Spectrophotometer. cDNA was synthesized by using a Verso cDNA Synthesis kit and the manufacturer’s instructions were followed. 600 ng of RNA was used for the synthesis of cDNA in a total volume of 50 μl. One to two microliters of cDNA templates were used for real-time PCR by using a SYBR Green qPCR master Mix kit in a Step One Plus Real-time PCR system. Each sample was evaluated in triplicate and the CT values were averaged before being subjected to normalization to GAPDH. All primer sequences used for qPCR are provided in Table 3. For cortical and hippocampal tissues as well as for purified microglia, sample sizes of *n* = 6 (three M, three F) were used, and data are presented as an average.

**Table 3:** Primers for qPCR

Gene	Sense (5'−3')	Anti-sense (3'−5')	Species
TNF-α	ATGGCCTCCCTCTCATCAGT	GTTTGCTACGACGTGGGCTA	Mouse
IL-1β	CGCAGCAGCACATCAACAAG	GTGCTCATGTCCTCATCCTG	Mouse
IL6	ACCAGAGGAAATTTTCAATAGGC	TGATGCACTTGCAGAAAACA	Mouse
IFN-α	GGACTTTGGATTCCCGCAGGAGAAG	GCTGCATCAGACAGCCTTGCAGGTC	Mouse
IFN-β	TCCGAGCAGAGATCTTCAGGAA	TGCAACCACCACTCATTCTGAG	Mouse
IFN-γ	GCTCTGAGACAATGAACGCT	AAAGAGATAATCTGGCTCTGC	Mouse
iNOS	TGGAGCGAGTTGTGGATTGTC	CCAGTAGCTGCCGCTCTCAT	Mouse
GAPDH	GACAACTTTGGCATTGTGG	ATGCAGGGATGATGTTCTG	Mouse

**Table 4:** Primers for genotyping

Gene	Sense (5'−3')	Anti-sense (3'−5')	Species
Cx3cr1 Cre+/−	TTGCCTGCATTACCGGTCGAT	GATCCTGGCAATTTCGGCTAT	Mouse
ATG7 flox - 373 bp	ACAGTGCACATCCTGTTCCA	CCAAAGGAAACCAAGGGAGT	Mouse
ATG7 flox - 243 bp	GGACTTGTGCCTCACCAGAT	CTCGTCACTCATGTCCCAGA	Mouse
TSC1 flow	GTCACGACCGTAGGAGAAGC	GAATCAACCCCACAGAGCAT	Mouse

### Purification of microglia from adult mouse brain

Animals were sedated with pentobarbital (100 mg/kg, i.p.) and transcardially perfused with 30 ml of 1× PBS. Mouse brains were dissected and dissociated in 1 ml ice-cold serum-free medium (DMEM/F12 + 1% pen/strep + 4.5 mg/ml glucose) and 3-ml dissociation medium (DMEM/F12 + 1 mg/ml papain + 1.2 unit/ml dispase II + 20 units/ml DNase I) per brain. Dissociated brain tissue solution was incubated at 37°C for 15 min, and the solution was neutralized immediately with 3 ml of neutralizing medium (DMEM/F12 + 10% FBS + 4.5 mg/ml glucose). The suspension was further dissociated by aspirating up-and-down with a 1 ml volume pipette and filtered through a 30-μm filter. Myelin was removed by adding 8 ml of 70% Percoll into the dissociated brain tissue solution. The tube containing the tissue solution was subjected to centrifugation for 10 min at 800 × *g* at room temperature in a brake-free condition. At this point, cells were pelleted and resuspended in 500 μl of FACS buffer (1% BSA, 0.1% sodium azide, and 2 mm EDTA in 1× PBS; pH 7.5). Cells were centrifuged at 500 × *g* for 5 min at 4°C and the pelleted cells were resuspended in 50 μl of FACS buffer; 1 μl of CD16/32 antibody was added to the cells to block Fc receptors and the suspension was incubated on ice for 10 min. The cells were then incubated with primary CX3CR1-PE conjugated antibody for 15 min at 4°C according to the manufacturer’s instructions. The cells were then washed with 500 μl of FACS buffer and centrifuged at 500 × *g* for 5 min at 4°C. The pelleted cells were resuspended in 80 μl of FACS buffer, and 20 μl of anti-PE microbeads were added. The cells were incubated for 20 min in the dark at 4°C, followed by washing in 500 μl of FACS buffer and centrifuged for 5 min at 500 × *g* at 4°C. Washed cells were resuspended in 500 μl of FACS buffer and passed through a magnetic Miltenyi MS column. The column was washed three times with 500 μl of FACS buffer and then removed from the magnetic field. At this point, microglial cells remain in the MS column and need to be forced out with a plunger. Cells were eluted out of the MS column according to the manufacturer’s instructions. Eluted cells were pelleted by centrifugation at 800 × *g* for 10 min at 4°C. FACS buffer was then removed and replaced with TRIzol for RNA extraction.

### Brain tissue collection for immunohistochemistry

Animals were injected with pentobarbital (100 mg/kg, i.p.) for anesthesia, and were transcardially perfused with 30 ml of 1× PBS followed by 30 ml of 4% paraformaldehyde in 1× PBS (PFA). Brains were postfixed further for at least 48 h in PFA and were cyroprotected in 30% sucrose for an additional 48 h before sectioning. Cryoprotected brains were then embedded in Neg-50 (Thermoscientific, 6502) frozen sectioning medium, and 35-μm coronal sections were cut using a Leica cryostat. Sectioned brain tissues were washed with 1× PBS and blocked with specific blocking buffer for immunohistochemistry analysis.

### Immunohistochemistry, acquisition of images, and image analysis

The following protocol was used for immunostaining tissue sections with anti-Iba1 (Wako, 019–19 471), anti-CD68 (Bio-Rad, MCA1957), anti-GFAP (Millipore, AB5541), anti-NeuN (Cell Signaling, D4640) antibodies. Comparable sections of ATG7KO and littermate control mice were chosen for staining. Selected sections were put on a slide and dried for 5 min. The sections were then washed with 1× PBS three times, 5 min each. The sections were then blocked and permeabilized with blocking buffer (0.3% Triton X-100 and 10% BSA in 1× PBS) for at least 1 h at room temperature. Then antibodies were diluted in blocking buffer and the sections were incubated with diluted antibodies overnight at 4°C. The following morning, the sections were washed with 1× PBS three times, 5 min per wash. Fluorophore-conjugated secondary antibodies were diluted in blocking buffer. The sections were incubated with secondary antibody for 2 h at room temperature and then washed with 1× PBS twice, 5 min per wash, then counterstained with DAPI (Sigma-Aldrich, D9542-1MG) diluted in 1× PBS for 5 min at room temperature, and then washed three times, 5 min per wash. Fluoromount G (SouthernBiotech, 0100-01) was overlaid on top of the brain sections and the slides were sealed with nail-polish. A confocal microscope (Zeiss LSM 880) was used to acquire images. A 25× water lens was used to collect images. To collect full-scale images, bounding grids were created. The grid was chosen to have the cortical layer directly above the hippocampus as well as the entire hippocampus. The z-layer was also added for image acquisition and a 15-μm-thick z-layer was chosen to image. The z-layer was chosen to have the maximum intensity. Images were taken in intervals of 1-μm thickness. Following acquisition, czi files were processed for airyscan processing, stitching of tiles, and to have maximum intensity projection. This was done by using ZEN Black 2.3. TIF format images were downloaded using ZEN Blue 2.3. For cell counting, ImageJ software was used and the “analyze particles” option was used to count cells. Data are presented to show cells per 10^5^ μm^2^. The sample size is six (three Males, three Females) for both ATG7KO mice and littermate controls.

Myelination was evaluated with anti-myelin basic protein (MBP; BioLegend, 808401), anti-proteolipid protein (PLP; Abcam, AB28486), and anti-myelin ODC glycoprotein (MOG; Millipore, MAB5680) antibodies, with a slightly modified protocol. Briefly, brain sections were stained in a free-floating condition (to have maximum antibody penetration) in a 96-well plate. The sections were incubated with blocking buffer (5% FBS and 2% Triton X-100 in 1× PBS) for 2 h at room temperature. Then, primary antibodies were diluted in the same blocking buffer and the sections were incubated with diluted antibodies for 2 h at room temperature. Following primary antibody incubation, the brain sections were washed using 1× PBS three times, 5 min per wash. After the PBS washes, secondary antibodies were diluted in the same blocking buffer and incubated for 2 h at room temperature, followed by washing and then counterstaining with DAPI (Sigma-Aldrich, D9542-1MG). Brain sections were mounted with Fluoromount G (SouthernBiotech, 0100-01). An epifluorescence microscope (Zeiss Imager M2; Neurolucida 2018 by MBF Bioscience) was used to acquire images. A 20× air lens was used to collect images. To collect full-scale images, a contour containing the entire brain section was drawn, and the brain sections were focused at multiple locations to have the entire section focused. For imaging intensity analysis, the “measure” option of ImageJ software was used to quantify staining intensity. Data are presented as percentage of wild-type. Three comparable sections were stained and imaged for each animal, and thus 18 images in total were imaged and quantified for each group.

For staining with anti-Olig2 (Millipore, AB9610) and anti-CC-1 (CalBioChem, OP80-100UG) antibodies, tissue sections were first processed via antigen retrieval. Antigen retrieval was achieved by heating 10 mm sodium citrate buffer (pH 6) in a container to boiling. The container was then removed from the heat source and glass slides containing the tissues were submerged in the warm buffer. The container was then put on a shaker with mild shaking. The slides were incubated for 20–25 min. Postantigen retrieval, tissue slices were washed with 1× PBS three times, 5 min each. Then, tissue slices were incubated with blocking buffer (2% goat serum + 1% Triton X-100 in 1× PBS) for at least 2 h at room temperature. The tissues were incubated with diluted primary antibodies in the same blocking buffer at 4°C overnight. After washing to remove the unbound primary antibody, the sections were incubated with secondary antibody for 2 h at room temperature, followed by washing, counterstaining, and mounting in Fluoromount G (SouthernBiotech, 0100-01). A confocal microscope (Zeiss LSM 880) and 25× water lens was used to collect images. To collect full-scale images, bounding grids were created. The grid was chosen to have the cortical layer directly above the corpus callosum and striatum. The z-layer was also added for image acquisition and a 15-μm thick z-layer was chosen to image. The z-layer was chosen to have the maximum intensity. Images were taken at intervals of 1-μm thickness. Following acquisition, czi files were processed for airyscan processing, stitching of tiles, and to have maximum intensity projection. In total, 18 images were acquired and quantified for each group. For cell counting of Olig2+/CC1+ cells, ImageJ software was used and the “analyze particles” option was used to count cells. Data are presented to show cells per 0.5 mm^2^.

The nodes of Ranvier (red) were positively stained for sodium channel using anti-Na_V_1.6, whereas the paranodes (green) that flank the nodes of Ranvier were labeled with anti-CASPR antibody. For tissue section staining with anti-Na_V_1.6 (Alomone labs, ASC-009) and anti-CASPR (Neuromab, Clone K65/35, 75-001) antibodies, the tissue sections were first put through antigen retrieval as described above, followed by blocking and permeabilization with blocking buffer (5% goat serum and 0.5% Triton X-100 in 1× PBS) for at least 2 h at room temperature. Tissues were then incubated with primary antibodies diluted in the same blocking buffer for 48 h at 4°C, followed by incubation with the secondary antibodies. Confocal images were acquired with a 63× oil lens. Z-stack images of 4 μm thick were taken at 0.20-μm intervals. Images were processed by using ZEN Black 2.3. For each animal, one section was stained and three different single images were taken for the cortical and corpus callosum regions. The length of the nodes of Ranvier was quantified using Imaris software. Maximum Intensity-projected czi images that were acquired from confocal microscopy were uploaded to the Imaris software. The length of the nodes was calculated automatically. The list of all sizes of node length calculated from Imaris was downloaded and used to calculate the average length and length distribution.

### Primary microglia culture

One- or 2-d-old pups were genotyped and their forebrains were dissected in dissection buffer (5% FBS in 1× PBS). The brains were then minced with sterilized razor blades and filtered through a sterile 40-μm filter. Cell suspensions were then pelleted by centrifuging for 5 min at 300 × *g*. The cells were then resuspended in 15 ml of culture medium (DMEM + 10% FBS + 1% pen/strep) and plated in a 75-mm^2^ flask. Two days after plating, M-CSF was added to the culture medium at 5 ng/ml. The culture medium was replaced every 2 d, and cells were allowed to grow for 12–14 d postseeding. To harvest microglia, flasks were shaken at 125 rpm for 4–5 h at 37°C. Detached microglia were then collected, pelleted by centrifuging for 5 min at 300 × *g*. Cells were resuspended in culture medium, counted, and plated in poly-D-lysine-coated 12-well plates or 35-mm dishes. Microglia cells were allowed to attach to the plate surface for 1 h at 37°C. Following 1 h of incubation, unattached cells were washed away. Cells in 35-mm dishes were allowed to grow for 2–3 d and used for the in vitro phagocytosis assay.

### *In vitro* phagocytosis assay

Primary microglial cells were cultured in 35-mm dishes according to the protocol described above. pHrodo Green zymosan bioparticles, dissolved at 0.5 mg/ml in phenol red-free DMEM, were used for this assay. Cell culture medium containing phenol red was removed immediately before live-cell imaging and was replaced with 100 μl of phenol red-free DMEM containing pHrodo Green zymosan bioparticles. The microscope-stage incubator was set at 37°C and the CO_2_ level was set at 5%. A confocal microscope (Zeiss LSM 880) was used to acquire images. A 63× oil lens was used to collect images. A series of images was collected in 60-s intervals for 60 min. Acquired czi images were processed by using ZEN Blue 2.3. A sample size of *n* = 4 was used for each mouse type.

### Seizure induction via pilocarpine

Pilocarpine was used to induce status epilepticus (SE). Mice 8–10 weeks of age were weighed and intraperitoneally injected with methyl scopolamine at 1 mg/kg in 0.9% NaCl. Animals were allowed to rest and roam around in their cages for 10 min to allow the scopolamine to take effect by blocking peripheral cholinergic receptors. Pilocarpine was dissolved in 0.9% NaCl and injected intraperitoneally into each mouse at a dose of 200 mg/kg for the first dose; the subsequent doses were applied at 15-min intervals at 50 mg/kg until each animal began to show seizure behaviors considered to be stage 4 or 5 of the Racine scale. Animals were allowed to have seizures for 4 h before being injected with Diazepam (5 mg/kg; dissolved in 12% ethanol) to terminate the seizures. Animals were kept on a heat-pad to minimize suffering and to allow for a higher yield. For sample sizes, 23 littermate controls (10 M, 13 F) and 22 ATG7KO mice (12 M, 10 F) were injected according to the protocol above.

### Seizure induction via kainic acid

Animals were anesthetized by using 4% isoflurane vapor, and anesthesia was confirmed by pinching a toe and looking for an absence of reflex. 100 μl of bupivacaine was injected under the forebrain skin before proceeding with surgery. Eye lubricant was also applied to prevent the eyes from drying out. Following anesthesia, animals were stereotaxically injected with 100 nl of kainic acid (20 or 50 μm). Animals were injected at the dorsal hippocampus region and the following coordinates from bregma were used: anteroposterior −2, mediolateral +1.5, dorsoventral −2. After the injection, the syringe was kept in for an additional 30–60 s to avoid backflow of the volume. Animals were returned to their home cage and their status of seizure development was monitored. For sample sizes, six littermate controls (three M, three F), and six ATG7KO (three M, three F) mice for each of the 20 and 50 μm experiments were injected according to the protocol above.

### Seizure induction via kindling

The surgical procedure described above was conducted to implant electrodes in the amygdala of each animal. The following coordinates were used: anteroposterior −1.2, mediolateral +3.1, dorsoventral −3.7. Two additional recording electrodes were implanted epidurally on the cortex on both the ipsilateral and contralateral sides. All electrodes were fixed with dental cement. Mice were allowed to recover for at least 7 d postsurgery before being subjected to kindling. Mice were kept in their home cages and were allowed to roam freely during the kindling procedures. Mice were hooked to a Grasso S88 stimulator device. The kindling procedure was adapted from Morales et al. (2014). To identify the after-discharge threshold (ADT), current intensity was started at 10 μA and gradually increased by a factor of 1.25 every 10 min until the appearance of the first after-discharge, which is defined as electrical waves between 1–8 Hz and lasting a minimum of 5 s. Stimulus intensity was 1× ADT. Mice were subjected to kindling for three consecutive days, and each day the animals received 10 stimulations with 20-min intervals between stimulations. Stimulations were 10 s long and the train frequency was 50 Hz (biphasic). After concluding the tests, mouse brains were fixed and sectioned for cresyl violet staining for histologic verification of electrode placement. Animals with a correct electrode placement in the right basolateral amygdala were used in subsequent data analyses.

### Behavior tests

Animal behavioral tests were performed in the Behavior Core of Albany Medical College. The animals were between eight and 10 weeks of age at the time of testing. The open field test (OFT) was used to measure the motor activity of animals by measuring the distance the animals traveled. Mice were brought into the OFT room at least 30 min before the OFT to allow them to acclimate to the room/environment. The apparatus that was used is a white cubical container divided into four 50 cm^2^ chambers, which allowed four mice to be tested at once. OFT was conducted under a dim, red light environment (50 lux) to allow the mice to roam and explore freely and their motor activity to be measured. ANY-maze 5.3 software was used to collect data; the program collected data on total distance traveled as well as time spent moving/average speed. The experiment lasted for 5 min for each animal and the OFT apparatus was cleaned with 70% ethanol and allowed to dry in between testing different animals. Sample sizes of 23 littermate controls (10 M, 13 F) and 22 ATG7^Cx3cr1-creCKO^ mice (12 M, 10 F) were tested. For rotarod test, mice were brought into the rotarod testing room at least 30 min before the test to allow them to acclimate to the room/environment. The test was conducted for five consecutive days and the tests were given at the same time every day. For the training period, animals were placed on the rotarod and trained at a fixed speed of 4 rpm. Then the animals were returned to their home cage for at least 10 min, and this step was repeated another two times. In between testing different animals, the rotarod was wiped and cleaned with 70% ethanol, and fecal pellets were removed. For the testing period, the rotarod was set to accelerate every 20 s by 1 rpm and the initial speed was 4 rpm. The exact speed and time a mouse fell from the rod was recorded. A mouse was given three trials per day and the per-day average was used to calculate the data.

### Statistical analysis

All data collected and presented were subjected to statistical analysis using GraphPad Prism7 software or estimation stats (free online version). Appropriate tests were applied for comparisons between control and ATG7KO mice. Estimation stats was used to test the significance of differences between the control and ATG7KO groups. Two-way ANOVA with repeated measures was used for the comparison of the time course for the latency to fall in the rotarod test between the control and ATG7KO groups. Data are shown as mean ± SEM. Log-rank (Mantel–Cox) test was used for statistical analysis of survival rate; *p* < 0.05 was considered to be significant All key resources related to the experimental procedures are listed in Tables 1–Tables 5.

**Table 1:** Antibodies

Product	Vendor	Catalog	Dilution	RRID
Anti-MOG	Millipore	MAB5680	IHC: 1:200 WB: 1:1000	AB_1587278
Anti-MBP	BioLegend	808401	IHC: 1:1000 WB: 1:1000	AB_2564741
Anti-CC-1	CalBioChem	OP80-100UG	IHC: 1:300	AB_2057371
Anti-Olig2	Millipore	AB9610	IHC: 1:200 WB: 1:2000	AB_570666
Anti-PLP	Abcam	Ab28486	IHC: 1:500 WB: 1:1000	AB_776593
Anti-p62	Cell Signaling	23214S	IHC: 1:400 WB: 1:1000	AB_2798858
Anti-NeuN	Cell Signaling	D4G4O	IHC: 1:1000	AB_2651140
Anti-Iba1	Wako	019-19741	IHC: 1:400	AB_839504
Anti-CD68	Bio-Rad/AbD Serotec	MCA1957	IHC: 1:400	AB_322219
Anti-GFAP	Millipore	AB5541	IHC: 1:300	AB_177521
Anti-GAPDH	Cell Signaling	5174S	WB: 1:1000	AB_10622025
Anti-mouse CD16/32	BioLegend	101302	Microglia purification: 1:50	AB_312801
PE anti-mouse CX3CR1	BioLegend	149006	Microglia purification: 1:50	AB_2564315
Anti-CASPR	Neuromab	Clone K65/35, 73-001	IHC: 1:200	AB_10671175
Anti-Na_V_1.6	Alomone Labs	ASC-001	IHC: 1:500	AB_2040003
Anti-mouse IgG, HRP conjugated	Cell Signaling	7076S	WB: 1:3000	AB_330924
Anti-rabbit IgG, HRP conjugated	Cell Signaling	7074S	WB: 1:3000	AB_2099233
Goat anti-Rabbit IgG (H + L) secondary antibody Alexa Fluor 488 conjugate	ThermoFisher	A-11034	IHC: 1:400	AB_257621
Goat anti-rabbit IgG (H + L) highly cross-adsorbed secondary antibody, Alexa Fluor 568	ThermoFisher	A-11036	IHC: 1:400	AB_10563566
Goat anti-mouse IgG (H + L) highly cross-adsorbed secondary antibody, Alexa Fluor 488	ThermoFisher	A-11001	IHC: 1:400	AB_2534069
Goat anti-mouse IgG (H + L) highly cross-adsorbed secondary antibody, Alexa Fluor 568	ThermoFisher	A-11031	IHC: 1:400	AB_144696
Goat anti-rat IgG (H + L) cross-adsorbed secondary antibody, Alexa Fluor 555	ThermoFisher	A-21434	IHC: 1:400	AB_2535855
Goat anti-chicken IgY (H + L) cross-adsorbed secondary antibody, Alexa Fluor 488	ThermoFisher	A-11039	IHC: 1:400	AB_142924
Goat anti-guinea pig IgG (H + L) cross-adsorbed secondary antibody, Alexa Fluor 568	ThermoFisher	A-11075	IHC: 1:400	AB_141954

**Table 2:** Reagents and other materials

Product	Vendor	Catalog no.	RRID
Neg-50	ThermoScientific	6502	
Fluoromount G	SouthernBiotech	0100-01	
DAPI	Sigma-Aldrich	D9542-1MG	
DMEM	Corning	10013-CV	
Phenol red-free DMEM	Life technologies	31053-028	
DMEM/F12	Gibco	11039-021	
Mouse M-CSF	Shenandoah	200-08	
Percoll	GE Healthcare	17-0891-01	
Papain	Sigma	P3125	
Dispase II	Stemcell	07913	
DNase I	Sigma	D4263	
Bovine serum albumin	Sigma	A7030	
Fetal bovine aerum	Atlanta Biologicalalals	S11150H	
Penicillin/streptomycin	Cellgro	30-008-CI	
Poly-D-lysine	Sigma	P6407-5MG	
PowerUP SYBR Green Master Mix	ThermoFisher	A25777	
TRIzol	Life Technologies	15596018	
Verso cDNA synthesis kit	ThermoFisher	AB1453B	
Pierce BCA Protein Assay kit	ThermoScientific	23225	
Chemiluminescent Substrate	ThermoScientific	34580	
pHrodo Green Zymosan Bioparticles Conjugate for Phagocytosis	ThermoFisher	P35365	
Falcon 40 μm Cell Strainer	Corning	352340	
Preseparation Filters (30 μm)	Miltenyi	130-041-407	
MS Columns	Miltenyi	130-042-201	
Pilocarpine	Sigma	P6503	

**Table 5:** Software

Product	Developer	Webpage
ImageJ	NIH	https://imagej.nih.gov/ij/
GraphPad Prism 7.0	GraphPad Software	https://graphpad.com
ZEN Black	Zeiss	https://zeiss.com
ZEN Blue	Zeiss	https://zeiss.com
Sirenia Acquisition	Pinnacle Technologies, Inc.	https://www.pinnaclet.com/sirenia.html
Neurolucida	MBF Bioscience	https://mbfbioscience.com
Imaris	Bitplane	https://imaris.oxinst.com/

## Results

### ATG7KO mice display increased seizure susceptibility and high mortality from seizures

**Figure 1.**
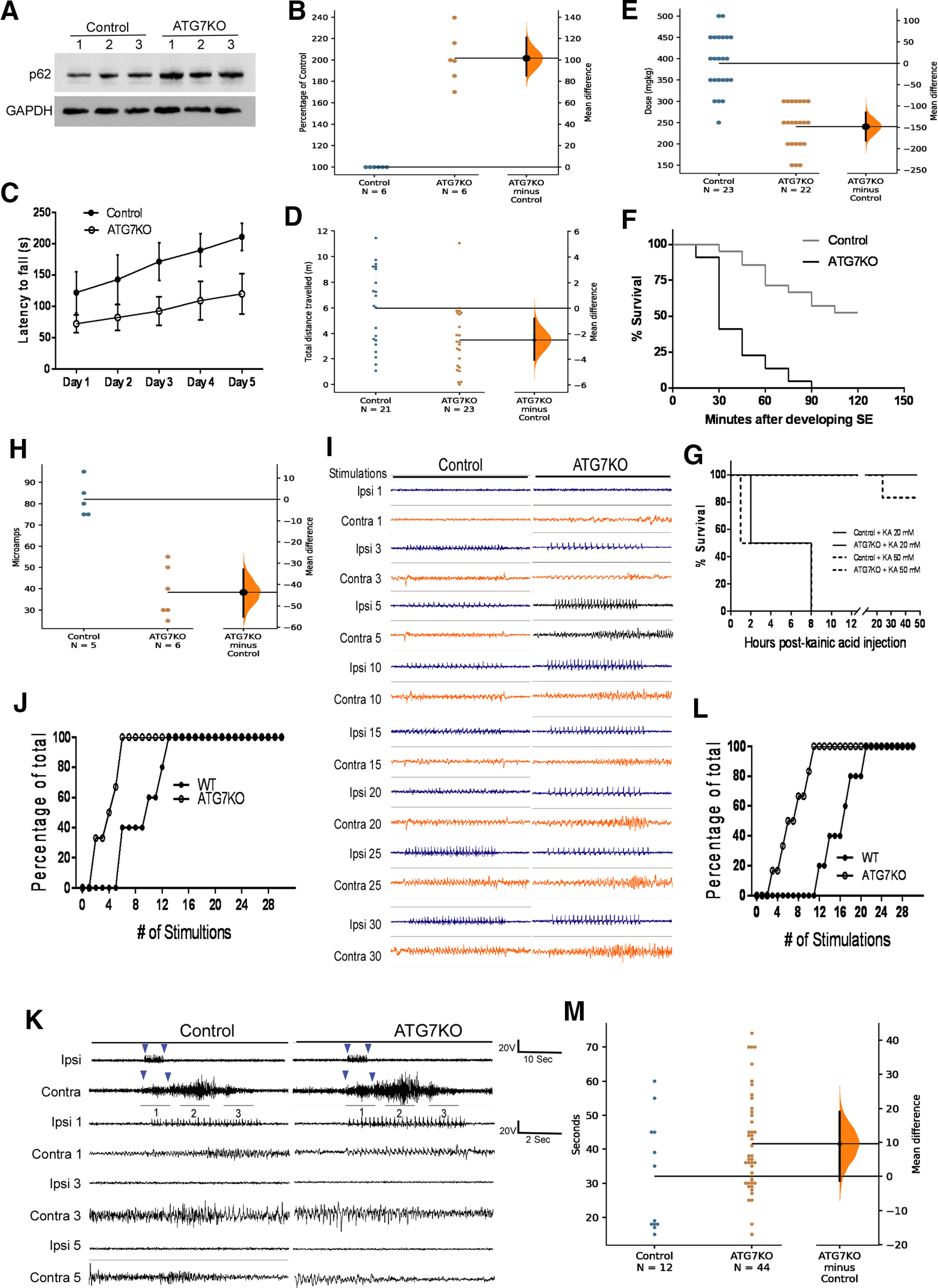
Impact of deletion of ATG7 in microglia on seizure susceptibility. ***A***, Western blot analysis of p62 in ATG7KO mice and their littermate controls. ***B***, Quantification of p62 protein levels in ATG7KO mice and their littermate controls; *n* = 6 (3 M, 3 F). The unpaired mean difference (hereafter referred to as UMD) between control and ATG7KO is 1.02e+02 (95.0%CI 85.1, 1.21e+02). The *p* value of the two-sided permutation *t* test (hereafter referred to as *p*_t_) is 0.0008. ***C***, Quantification of latency to fall in the rotarod test in 21 littermate control (11 M, 10 F) and 23 ATG7KO mice (10 M, 13 F). Two-way ANOVA with repeated measures; group effect: *F*_(4,110)_ = 63.46, *p* < 0.0001; time effect: *F*_(4,110)_ = 294.1, *p* < 0.0001; interaction: *F*_(4,110)_ = 3.104, *p* = 0.0184 (CI = 95%). ***D***, Quantification of total distance traveled in the OFT in 21 littermate control (11 M, 10 F) and 23 ATG7KO mice (10 M, 13 F). UMD: −2.48 (95.0%CI −4.05, −0.791); *p*_t_ = 0.0068. ***E***, Pilocarpine doses to induce SE in in 23 control (10 M, 13 F) and 22 ATG7KO (12 M, 10 F) mice. UMD: −1.48e+02 (95.0%CI −1.82e+02, −1.15e+02); *p*_t_ = 0.0. ***F***, Percentage of control and ATG7KO mice surviving SE induced by pilocarpine in 23 control (10 M, 13 F) and 22 ATG7KO (12 M, 10 F) mice. Log-rank (Mantel–Cox) test: *p* < 0.0001. ***G***, Percentage of control and ATG7KO mice surviving SE induced by kainic acid (0.5 μl of 20 and 50 μm) injected into the dorsal dentate gyrus of the hippocampus; *n* = 6 (3 M, 3 F). Log-rank (Mantel–Cox) test: *p* = 0.0009 between control+KA 20 mm and ATG7KO+KA 20 mm; *p* = 0.0009 between control+KA 50 mm and ATG7KO+KA 50 mm. ***H***, After-discharge threshold in control and ATG7KO mice subjected to kindling. UMD: −43.7 [95.0%CI −55.2, −32.8]; *p*_t_: 0.0. ***I***, Representative epidural recordings of electrical activities in ipsilateral and contralateral sides. ***J***, Numbers of stimulations needed to elicit contralateral activities in control and ATG7KO mice. Two-way ANOVA; group effect: *F*_(30,30)_ = 7.336, *p* < 0.0001; number of stimulation effect: *F*_(1,30)_ = 14.43, *p* = 0.0007. ***K***, Representative EEG traces of animals that developed generalized seizures because of kindling. ***L***, Numbers of stimulations needed to trigger generalized seizures in control and ATG7KO mice. Two-way ANOVA; group effect: *F*_(30,30)_ = 5.3, *p* < 0.0001; number of stimulation effect: *F*_(1,30)_ = 26.28, *p* < 0.0001. ***M***, Average seizure duration in control and ATG7KO mice. UMD: 9.55 (95.0%CI −1.34, 19.0); *p*_t_ = 0.0506.

The ATG7KO line with ATG7 deletion in microglia was generated by crossing ATG7^f/f^ mice (McMahon et al., 2012) with the Cx3cr1-cre line that was recently demonstrated to be microglia specific (Zhao et al., 2019). Western blot analysis revealed that deletion of ATG7 results in accumulation of p62 (Fig. 1*A*,*B*), a marker of autophagy inactivity (Friedman et al., 2012; McMahon et al., 2012), confirming that autophagy activity is suppressed. Most ATG7KO mice are born without obvious abnormalities and can live for >12 months. None of the ATG7KO mice developed spontaneous seizures (data not shown). However, we observed two mice out of 43 that displayed marked locomotor impairment (data not shown). These two mice showed loss of postural control and hardly moved by the age of weaning, and they were humanely euthanized. Accordingly, we performed rotarod test and OFT on a cohort of ATG7KO mice to evaluate general motor behaviors. We observed that ATG7KO mice fell more easily from the rotarod onto the platform in rotarod tests and traveled shorter distances in OFTs compared with wild-type littermates (Fig. 1*C*,*D*). We next determined whether ATG7KO mice are more susceptible to developing spontaneous seizures in the pilocarpine model of temporal lobe epilepsy. Mice were treated with a single dose of pilocarpine at 200 mg/kg followed by 50 mg/kg every 10 min via intraperitoneal injection until they developed SE. The induction of SE in ATG7KO mice required less than half the dose needed in the control mice (Fig. 1*E*). In the littermate control group, over 50% of the mice survived 2 h of SE. However, ATG7KO mice became very sensitive to pilocarpine-induced SE. More strikingly, all ATG7KO mice died within 30–90 min after induction of SE (Fig. 1*F*). To exclude any potential systemic effect from pilocarpine treatment, we employed another SE model established using intracranial hippocampal injection of kainic acid (Fig. 1*G*). In this model, nearly 80% of the control mice survived SE. However, all ATG7KO mice died from seizures. These data suggest that ATG7KO mice are very susceptible to developing severe, generalized fatal seizures induced by pilocarpine or kainic acid.

We next employed an electric amygdala kindling model (Goddard et al., 1969; Sato et al., 1990). The seizures evoked in this model are moderate and brief, which helps to better evaluate the difference in seizure susceptibility between control and ATG7KO mice. We examined whether ATG7KO animals are prone to evoked seizures and whether after-discharges can readily propagate to the contralateral hemisphere, a process underlying the development of generalized seizures. We implanted a pair of electrodes into the right amygdala and two further electrodes epidurally on the ipsilateral and contralateral cortices for recording the propagation of electrical activities evoked by amygdala stimulation. We found that the intensity of electric currents required to elicit after-discharge in ATG7KO mice was ∼50% of that needed in control mice, consistent with increased neuronal excitability in the former (Fig. 1*H*). Animals were subjected to a rapid kindling protocol with 10 trains of stimulations with 20-min intervals daily for three consecutive days (Morales et al., 2014). The amygdala stimulation evoked an equally large amplitude of electrical activities in the ipsilateral cortex in both control and ATG7KO mice (Fig. 1*I*). Amygdala stimulation also evoked electrical activities in the contralateral cortex in all ATG7KO mice after four to five trains of stimulation (Fig. 1*J*). However, only very small-amplitude and short-lived electrical activities were evoked in the control animals during the 10 trains of repeated stimulations (Fig. 1*J*). Moreover, amygdala stimulation triggered the onset of stage four to five generalized behavioral seizures (Fig. 1*K–M*) in all ATG7KO mice within the first 10 trains of stimulation, whereas nearly double the number of stimulations was required in the control mice. These data suggest that seizure induction from amygdala stimulation as well as contralateral propagation of seizures is markedly increased in ATG7KO mice. Taken together, we demonstrated that ATG7KO mice display increased susceptibility to developing severe generalized seizures and high co-mortality.

### The impact of microglial autophagy deficiency on microglia, astrocytes, and neurons

**Figure 2.**
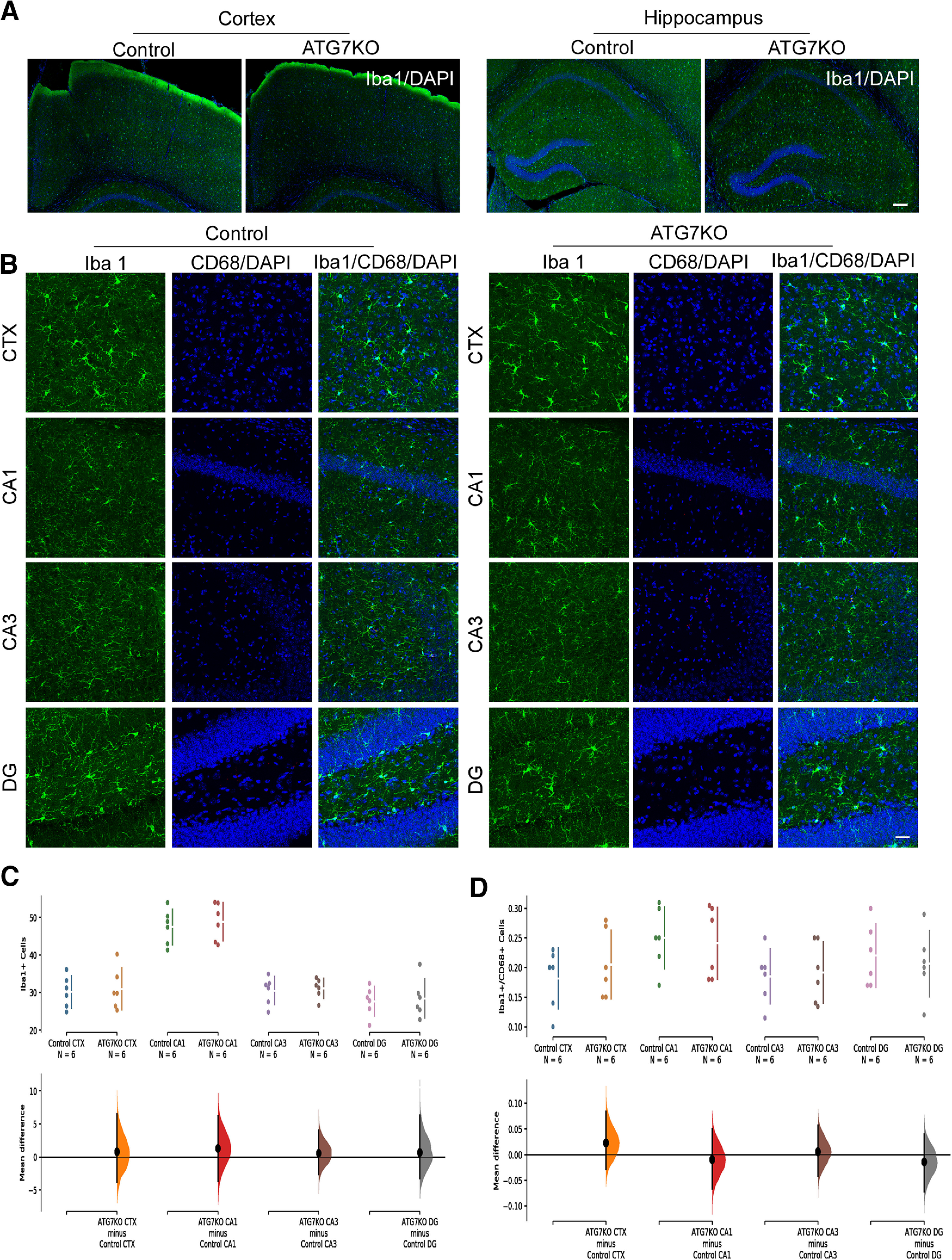
The impact of ATG7 deletion on microglia. ***A***, Full-montage images of Iba1^+^ cells in the cortical and hippocampal areas in control and ATG7KO mice. Scale bar: 200 μm. Green: anti-Iba1; blue: DAPI. ***B***, Confocal images (25×) of microglia from the cortical (CTX) and hippocampal CA1, CA3, and DG regions of control and ATG7KO mice. Scale bar: 20 μm. Green: anti-Iba1; red: anti-CD68; blue: DAPI. ***C***, Quantification of Iba1^+^ cells per 10^5^ μm^2^ (*n* = 6; 3 M, 3 F). CTX: UMD: 0.789 (95.0%CI −3.81, 6.49); *p*_t_ = 0.788. CA1: UMD: 1.35 (95.0%CI −3.65, 6.21); *p*_t_ = 0.62. CA3: UMD: 0.617 (95.0%CI −2.59, 4.02); *p*_t_ = 0.747. DG: UMD: 0.717 (95.0%CI −3.22, 6.28); *p*_t_ = 0.797. ***D***, Quantification of CD68^+^/Iba1^+^ cells per 10^5^ μm^2^ (*n* = 6; 3 M, 3 F). CTX: UMD: 0.0233 (95.0%CI −0.0283, 0.0833); *p*_t_ = 0.448. CA1: −0.009 (95.0%CI −0.0665, 0.0503); *p*_t_ = 0.786. CA3: UMD: 0.0065 (95.0%CI −0.0418, 0.057); *p*_t_ = 0.806. DG: UMD: −0.0133 (95.0%CI −0.0717, 0.04); *p*_t_ = 0.68.

**Figure 3.**
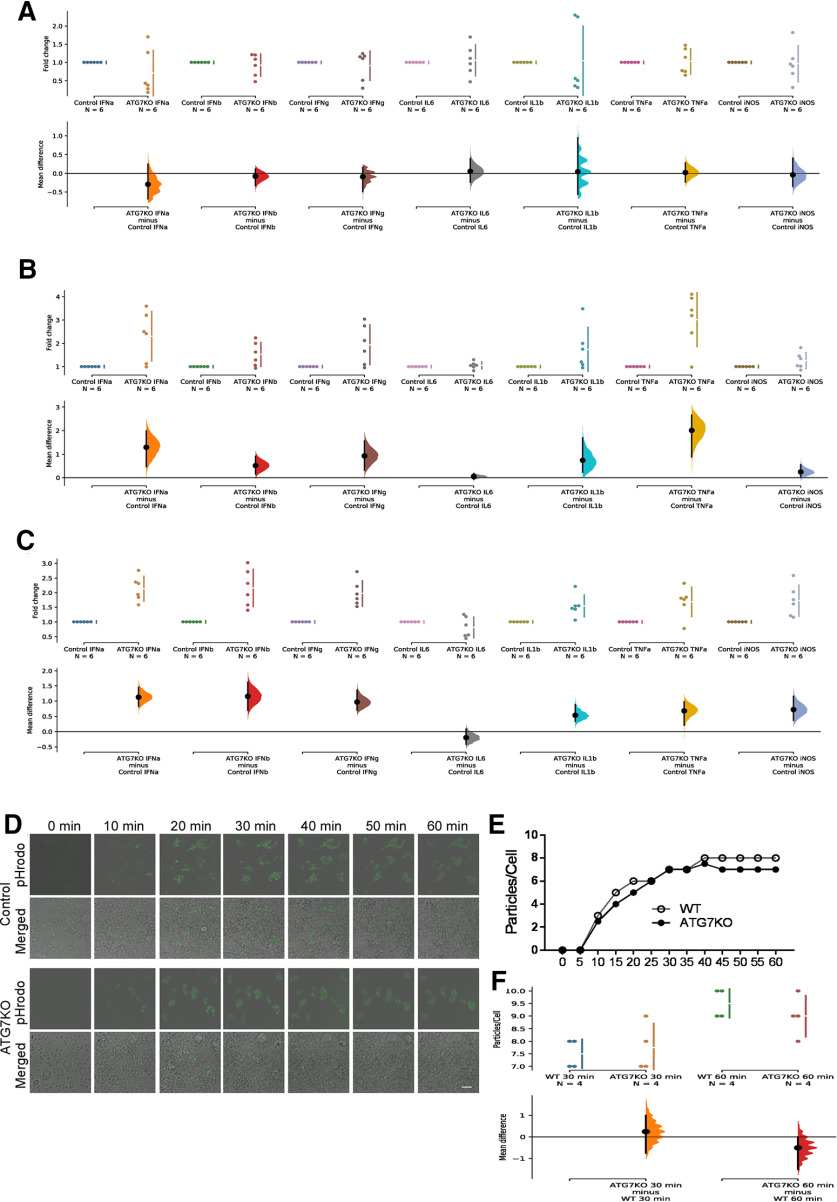
The impact of ATG7 deletion on microglial cytokines and phagocytosis. ***A***, Cytokine expression in cortical tissue samples from control and ATG7KO mice (*n* = 6; 3M, 3F). IFNa: UMD: −0.292 [95.0%CI −0.684, 0.249]; *p*_t_: 0.301. IFNb: UMD: −0.0762 [95.0%CI −0.329, 0.118]; *p*_t_: 0.527. IFNg: UMD: is −0.0877 [95.0%CI −0.486, 0.165]; *p*_t_: 0.561. IL6: UMS: 0.0582 [95.0%CI −0.241, 0.392]; *p*_t_: 0.753. IL1b: UMD: 0.0473 [95.0%CI −0.568, 0.949]; *p*_t_: 0.835. TNFa: UMD: 0.0262 [95.0%CI −0.233, 0.28]; *p*_t_: 0.864. iNOS: UMD: −0.0356 [95.0%CI −0.35, 0.404]; *p*_t_: 0.834. ***B***, Cytokine expression in hippocampal tissue samples from control and ATG7KO mice (*n* = 6; 3M, 3F). IFNa: UMD: 1.31 [95.0%CI 0.47, 1.99]; *p*_t_: 0.0154. IFNb: UMD: 0.525 [95.0%CI 0.153, 0.918]; *p*_t_: 0.0486. IFNg: UMD: 0.938 [95.0%CI 0.322, 1.56]; *p*_t_: 0.0148. IL6: UMS: 0.0553 [95.0%CI −0.0599, 0.174]; *p*_t_: 0.51. IL1b: UMD: 0.746 [95.0%CI 0.232, 1.7]; *p*_t_: 0.0142. TNFa: UMD: 2.01 [95.0%CI 0.878, 2.66]; *p*_t_: 0.0108. iNOS: UMD: 0.256 [95.0%CI 0.0246, 0.56]; *p*_t_: 0.118. ***C***, Cytokine expression in purified microglial samples of control and ATG7KO mice (*n* = 6; 3M, 3F). IFNa: UMD: 1.13 [95.0%CI 0.828, 1.45]; *p*_t_: 0.0. IFNb: UMD: 1.16 [95.0%CI 0.697, 1.62]; *p*_t_: 0.0. IFNg: UMD: 0.973 [95.0%CI 0.703, 1.36]; *p*_t_: 0.0. IL6: UMS: −0.192 [95.0%CI −0.419, 0.0818]; *p*_t_: 0.164. IL1b: UMD: 0.541 [95.0%CI 0.334, 0.891]; *p*_t_: 0.0. TNFa: UMD: 0.683 [95.0%CI 0.211, 0.977]; *p*_t_: 0.0138. iNOS: UMD: 0.724 [95.0%CI 0.366, 1.16]; *p*_t_: 0.0002. ***D***, Representative time series images (63×) showing in vitro uptake of pHrodo zymosan bioparticles (green) in microglia prepared from control and ATG7KO mice; Scale bar – 25 μm. ***E***, Representative time-course showing the number of bioparticles taken up. ***F***, Average particles per microglial cell at the 30- and 60-min time points (*n* = 4); 30 min: UMD: 0.25 [95.0%CI −0.75, 1.0]; *p*_t_: 0.365. 60 min: UMD: −0.5 [95.0%CI −1.5, 0.0]; *p*_t_: 0.112.

**Figure 4.**
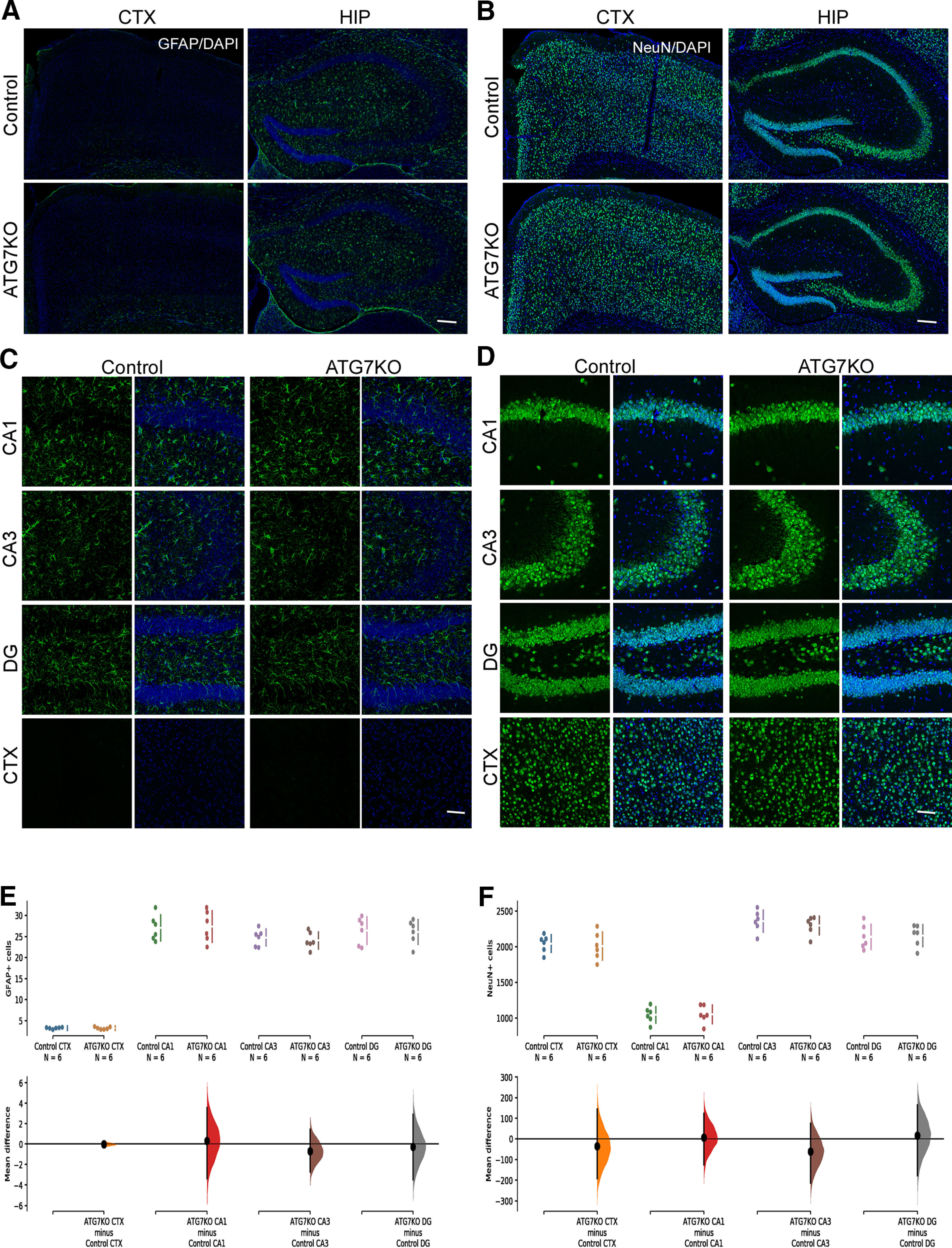
Impact of microglial ATG7 deletion on astrocytes and neurons. ***A***, Full-montage images of GFAP expression in the cortical and hippocampal areas from littermate control and ATG7KO mice. Green: GFAP; Blue: DAPI. Scale bar – 200 μm. ***B***, Full-scale images of NeuN expression in the cortical and hippocampal areas of littermate control and ATG7KO mice. Green: NeuN; Blue: DAPI. Scale bar – 200 μm. ***C***, Confocal images (25×) of astrocytes from the cortical (CTX) and hippocampal CA1, CA3 and DG regions of littermate control and ATG7KO mice. Green: GFAP; Blue: DAPI. Scale bar – 20 μm. ***D***, Confocal images of neurons from the cortical (CTX) and hippocampal CA1, CA3 and DG regions of littermate control and ATG7KO mice. Scale bar – 20 μm. ***E***, Quantification of GFAP^+^ cells per 10^5^ μm^2^. *n* = 6 (3 M, 3 F). CTX: UMD: −0.02 [95.0%CI −0.253, 0.26]; *p*_t_: 0.881. CA1: UMD: 0.3 [95.0%CI −3.35, 3.57]; *p*_t_: 0.886. CA3: UMD: −0.7 [95.0%CI −2.7, 1.43]; *p*_t_: 0.549. DG: UMD: −0.283 [95.0%CI −3.45, 2.9]; *p*_t_: 0.862. ***F***, Quantification of NeuN^+^ cells per 10^5^ μm^2^. *n* = 6 (3 M, 3 F). CTX: UMD: −36.0 [95.0%CI −1.91e+02, 1.44e+02]; *p*_t_: 0.708. CA1: UMD: 5.83 [95.0%CI −1.25e+02, 1.24e+02]; *p*_t_: 0.925. CA3: UMD: −61.7 [95.0%CI −2.12e+02, 75.5]; *p*_t_: 0.466. DG: UMD: 17.2 [95.0%CI −1.77e+02, 1.64e+02]; *p*_t_: 0.846.

We did not find any difference in the density of microglia in the cortex and hippocampus of ATG7KO mice compared with controls (Fig. 2*A–C*). CD68 is a lysosomal protein. The level of CD68 expression is nearly undetectable in microglia of normal brain, but is frequently induced following seizures and other neurologic disorders (Hopperton et al., 2018; Zhao et al., 2018). We did not detect any induction of CD68 in ATG7KO microglia (Fig. 2*B*,*D*). We next analyzed cytokine expression. There was no difference in the expression levels of TNFα, IL1β, IL6, IFNs, and iNOS in cortical lysates (Fig. 3*A*), and only a moderate elevation (∼2–3-fold) in hippocampal lysate or purified microglia (Fig. 3*B*,*C*). Microglia are the main phagocytotic cells (Zhao et al., 2018). We performed a phagocytosis analysis in cultured microglia prepared from ATG7KO and control mice. We did not detect any significant change in phagocytosis activity of ATG7-deficient microglia compared with the control microglia (Fig. 3*D–F*). Thus, microglial autophagy deficiency causes little, if any, change in density and morphology of microglia or phagocytotic activity. We next evaluated the impact of autophagy-deficient microglia on astrocytes and neurons in ATG7KO mouse brain. We did not observe any significant increase in astrocytes in the cortex and hippocampus (Fig. 4*A*,*C*,*E*), nor any effect on the density of neurons (Fig. 4*B*,*D*,*F*).

### Microglial autophagy deficiency leads to increased myelination and size of the nodes of Ranvier

**Figure 5.**
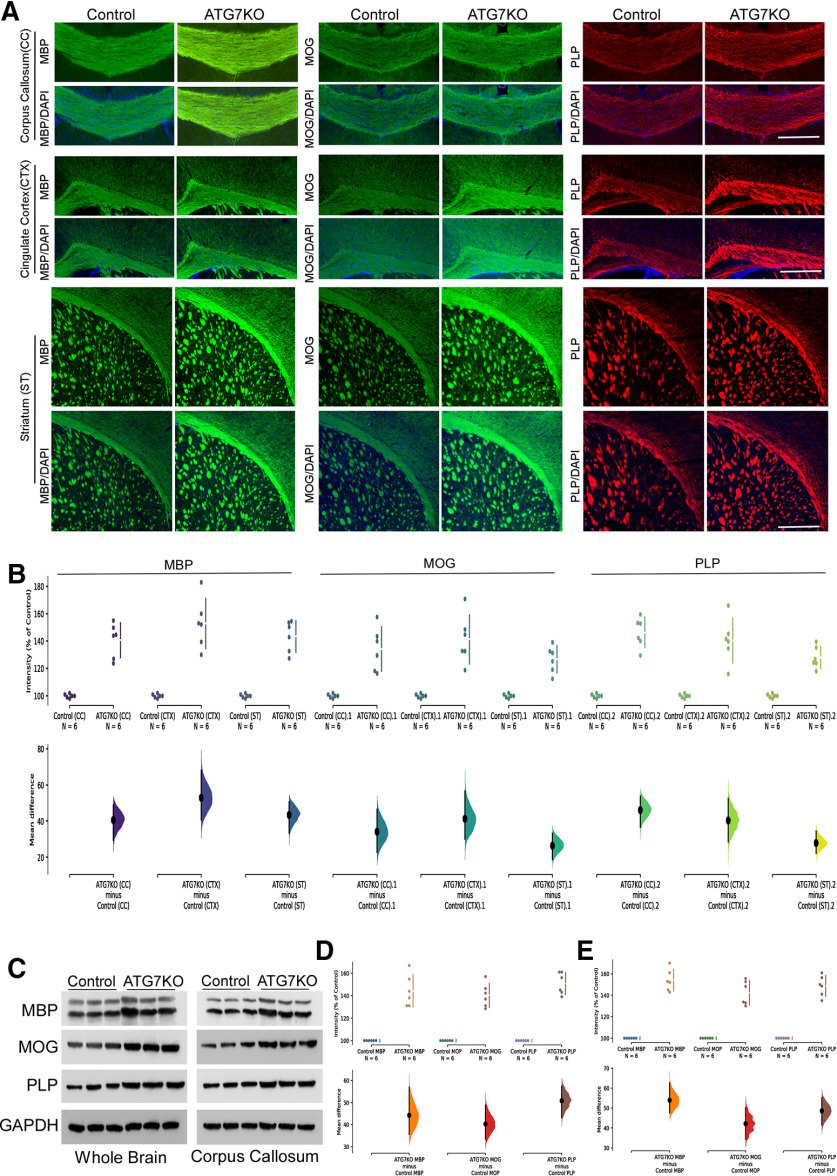
Impact of microglial ATG7 deletion on expression of myelination markers. ***A***, Representative images (20×) of corpus callosum (CC), cingulate cortex (CX) and striatum (ST) from littermate control and ATG7KO mice stained with MBP, MOG and PLP antibodies. ***B***, Quantification of MBP, MOG and PLP expression in control and ATG7KO mice; *n* = 6 (3 M, 3 F). MBP: Corpus callosum (CC): UMD: 40.6 [95.0%CI 29.5, 48.8]; *p*_t_: 0.0016. Cortex (CTX): UMD: 52.9 [95.0%CI 40.7, 68.0]; *p*_t_: 0.0. Striatum (ST): UMD: 43.5 [95.0%CI 33.5, 50.5]; *p*_t_: 0.0. MOG: Corpus callosum (CC): UMD: 34.2 [95.0%CI 23.0, 46.4]; *p*_t_: 0.0002. Cortex (CTX): UMD: 41.3 [95.0%CI 30.3, 56.5]; *p*_t_: 0.0. Striatum (ST): UMD: 26.6 [95.0%CI 18.8, 33.2]; *p*_t_: 0.0. PLP: Corpus callosum (CC): UMD: 46.2 [95.0%CI 36.9, 53.6]; *p*_t_: 0.0. Cortex (CTX): UMD: 40.5 [95.0%CI 28.6, 52.4]; *p*_t_: 0.0006. Striatum (ST): UMD: 27.9 [95.0%CI 22.5, 34.3]; *p*_t_: 0.0004. ***C***, Western blot analysis of MBP, MOG and PLP protein levels from whole brain and corpus callosum lysates; *n* = 6 (3 M, 3 F). ***D***, Quantification of MBP, MOG and PLP protein levels from whole brain. MBP: UMD: 44.3 [95.0%CI 35.5, 56.9]; *p*_t_: 0.0004. MOG: UMD: 40.3 [95.0%CI 33.4, 48.8]; *p*_t_: 0.0002. PLP: UMD: 50.8 [95.0%CI 43.2, 57.3]; *p*_t_: 0.0006. ***E***, Quantification of MBP, MOG and PLP protein levels from isolated corpus callosum lysates. MBP: UMD: 54.0 [95.0%CI 47.7, 62.7]; *p*_t_: 0.0. MOG: UMD: 42.2 [95.0%CI 34.3, 50.1]; *p*_t_: 0.0. PLP: UMD: 48.6 [95.0%CI 41.5, 55.2]; *p*_t_: 0.0.

Recent studies strongly suggest that microglia regulate ODC density and perhaps myelination (Miron, 2017; Wlodarczyk et al., 2017; Lloyd and Miron, 2019). Accordingly, we examined myelination markers in ATG7KO mice. Immunohistological analysis revealed a significant increase in MBP, along with markers of myelination (MOG and PLP) in the corpus callosum, cingulate cortex and striatum, regions where axons are heavily myelinated (Fig. 5*A*,*B*). Western blottings confirmed the increased levels of these myelination markers in ATG7KO mouse brains (Fig. 5*C–E*).

**Figure 6.**
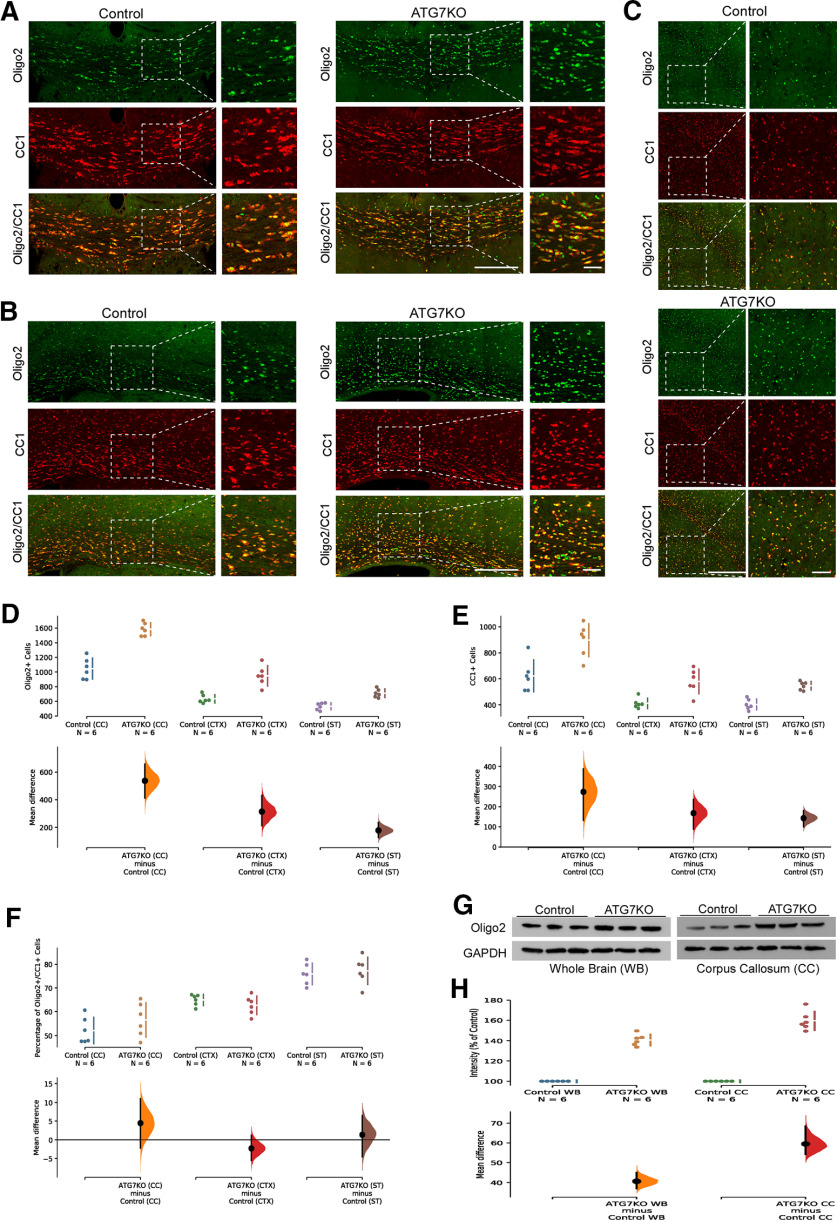
Impact of microglial ATG7 deletion on oligodendrocytes. ***A***–***C***, Representative confocal images (25×) of corpus callosum (CC) (***A***), cingulate cortex (CTX) (***B***) and striatum (ST) (***C***) from littermate control and ATG7KO mice stained with an oligodendrocyte marker (Olig2; green) and a marker of mature oligodendrocytes (CC-1; red). Scale bar – 20 μm, 5 μm. ***D***–***F***, Quantification of Olig2^+^ cells, CC1^+^ cells and Olig2^+^/CC1^+^ cells per 0.5 mm2; *n* = 6 (3 M, 3 F). Oligo2^+^ cells (***D***): Corpus callosum (CC): UMD: 5.38e+02 [95.0%CI 4.13e+02, 6.58e+02]; *p*_t_: 0.0. Cortex (CTX): UMD: 3.14e+02 [95.0%CI 2.14e+02, 4.3e+02]; *p*_t_: 0.0. Striatum (ST): UMD: 1.78e+02 [95.0%CI 1.27e+02, 2.36e+02]; *p*_t_: 0.0. CC1^+^ cells (***E***): Corpus callosum (CC): UMD: 2.74e+02 [95.0%CI 1.32e+02, 3.87e+02]; *p*_t_: 0.0046. Cortex (CTX): UMD: 1.68e+02 [95.0%CI 88.7, 2.36e+02]; *p*_t_: 0.0024. Striatum (ST): UMD: 1.43e+02 [95.0%CI 1.01e+02, 1.79e+02]; *p*_t_: 0.0. Olig2^+^/CC1^+^ cells (***F***): Corpus callosum (CC): UMD: 4.49 [95.0%CI −2.21, 10.9]; *p*_t_: 0.245. Cortex (CTX): UMD: −2.26 [95.0%CI −5.52, 1.15]; *p*_t_: 0.254. Striatum (ST): UMD: 1.39 [95.0%CI −4.52, 6.48]; *p*_t_: 0.636. ***G***, Western blot analysis of Olig2 protein levels in whole brain and isolated corpus callosum lysates. ***H***, Quantification of Olig2 protein levels in whole brain and isolated corpus callosum lysates *n* = 6 (3 m and 3 f). Whole brain: Oligo2: UMD: 40.7 [95.0%CI 36.9, 45.1]; *p*_t_: 0.0. Corpus callosum (CC): Oligo2: UMD: 59.5 [95.0%CI 54.1, 68.5]; *p*_t_: 0.0.

To understand how microglial deficiency of autophagy leads to increased myelination markers, we evaluated total ODCs and mature myelin-producing ODCs. Olig2 is expressed by ODCs in most stages of ODC development, whereas CC1 expression is more restricted to mature, myelin-producing ODCs. IHC revealed a significant increase in both Olig2-positive and CC1-positive populations in the corpus callosum, cingulate cortex, and striatum in ATG7KO mice (Fig. 6*A–F*). Western blottings also confirmed the increased levels of Olig2 in mouse brains (Fig. 6*G*,*H*). Together, these data suggest that suppression of microglial autophagy increases the amount of mature ODCs and myelination proteins.

**Figure 7.**
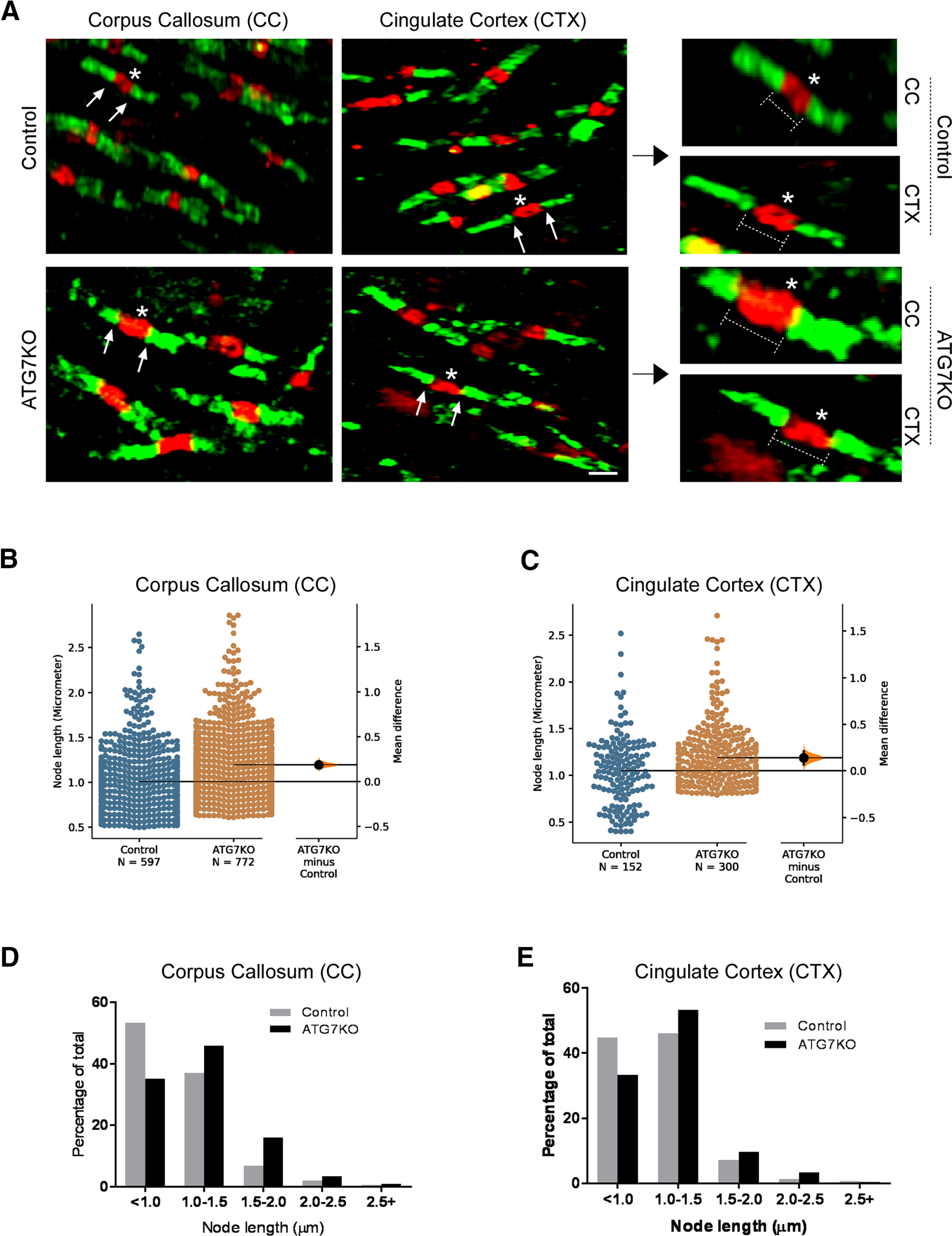
Microglial deletion of ATG7 changes the size of the nodes of Ranvier. ***A***, Representative confocal images (63×) of IHC staining for sodium channel Nav1.6 (red) and CASPR (green) in the corpus callosum (CC) and cortex (CTX) areas of littermate control and ATG7KO mice. Scale bar – 20 μm. ***B***, ***C***, Quantification of the average size of nodes in the CC (***B***) and CTX (***C***) in littermate control and ATG7KO mice. Data points were acquired from 6 pairs of animals (3 M, 3 F). Corpus callosum (CC): UMD: 0.188 [95.0%CI 0.148, 0.231]; *p*_t_: 0.0. Cingulate cortex (CTX): UMD: 0.141 [95.0%CI 0.0685, 0.211]; *p*_t_: 0.0. D, E, Distribution of the length of nodes in the corpus callosum (***D***) and cingulate cortex (***E***) of littermate control and ATG7KO mice.

The nodes of Ranvier mediate the stationary transmission of action potentials along axons. Recent studies reported that the size of the nodes of Ranvier is proportional to the speed of action potential transmission (Ford et al., 2015; Arancibia-Cárcamo et al., 2017). Lengthening of the node of Ranvier is strongly associated with altered function of the myelinated axon in several neurologic disorders (Arancibia-Cárcamo et al., 2017). Accordingly, we performed IHC to evaluate the nodes of Ranvier (using anti-sodium channel Na_V_1.6 antibody) and the paranodes (using anti-CASPR antibody; Arancibia-Cárcamo et al., 2017). We detected a shift toward longer nodes of Ranvier in ATG7KO mouse brain (Fig. 7*A–E*). Taken together, these data suggest that the microglial autophagy regulates homeostasis of ODCs and myelination of axons.

## Discussion

In the present study, we showed that ATG7KO mice with microglial autophagy deficiency are more susceptible to seizures as well as to developing severe generalized seizures resulting in death, compared with wild-type littermates. We further revealed that inhibition of autophagy in microglia disturbs ODC homeostasis in the CNS, leading to increased density of mature ODCs, elevated levels of myelination proteins, and increased size of the nodes of Ranvier. Our study revealed a critical role of microglial autophagy in regulating homeostasis of ODCs and seizure susceptibility.

### Role of autophagy in microglia

Autophagy activity is regulated according to changes in physiological conditions, including growth factors, nutrient bioavailability, stress, and many others (Levine and Klionsky, 2004). In general, autophagy acts downstream of mTOR. Our previous study demonstrated that excessive activation of microglial mTOR in TSC1 deficiency mice causes marked proliferation of microglia and significant morphologic changes (Zhao et al., 2018). We found that deletion of microglial ATG7 has little effect on the density and morphology of microglia. This is in consistent with observations reported in a recent study (Choi et al., 2020). Our data also suggest that basal autophagy activity does not likely contribute to the changes in microglial properties seen in TSC1 deficiency mice (Zhao et al., 2018).

Autophagy regulates the innate immune response of macrophages and microglia (Sanjuan et al., 2007; Cunha et al., 2018; Samie et al., 2018; Mathur et al., 2019). Deficiency of autophagy in macrophages exacerbates the inflammatory response and causes autoimmune diseases (Mathur et al., 2019). Inhibition of autophagy is an essential step leading to activation of microglia and induction of proinflammatory cytokines following LPS treatment (He et al., 2018). We found that under basal conditions, deficiency of autophagy leads to a very moderate increase in the levels of cytokines. It is unclear whether the mild elevation of cytokines is meaningful, and its biological significance needs to be determined in future studies. Microglia are the main phagocytotic cells in the CNS. Although previous studies suggest that autophagy regulates phagocytosis of microglia (Heckmann et al., 2019; Lee et al., 2019), the lack of effect on phagocytosis in our study may suggest the existence of both autophagy-dependent and -independent phagocytotic routes in microglia.

### Role of microglial autophagy in myelination

A large body of studies has revealed that mTOR signaling has a profound role in regulating the development of ODCs and myelination (Meikle et al., 2007; Carson et al., 2012; Lebrun-Julien et al., 2014; Wahl et al., 2014; Jiang et al., 2016; Beirowski et al., 2017; Figlia et al., 2017). However, all these studies focused on modifying mTOR signaling directly either in neurons, ODCs or Schwann cells. More recently, microglia were reported to regulate ODC development and myelination (Miron et al., 2013; Wlodarczyk et al., 2017). However, the exact mechanism by which microglia regulate ODC development and myelination remains to be elucidated. We found that inactivation of autophagy in microglia leads to increased numbers of mature ODCs and markers of myelination. Our results reveal a novel mechanism by which microglia act to maintain homeostasis of ODCs in the CNS. Microglial autophagy deficiency leads to an increased number of mature myelin-producing ODCs and levels of myelin markers in white matter structures, likely reflecting increased myelination. Future study will characterize the myelination structures, such as the thickness of the myelin sheath and the density of internodes along the axons. A recent study reported that the ATG7 gene in microglia plays a role in myelin degradation and clearance (Berglund et al., 2020). It will be interesting to determine whether compromised myelin degradation and clearance of ATG7-deficient microglia contribute to altered levels of myelin and myelination markers and ODC density.

The mechanism underlying the regulation of ODC homeostasis by microglia remains to be determined. It is conceivable that deletion of microglial ATG7 influences the expression of myelin proteins/myelination markers and the density of ODCs through many routes. ATG7 deletion could impose a direct effect on ODCs and their progenitors or act indirectly on other cells such as neurons and astrocytes. It is very hard if not impossible to differentiate these two possibilities at this time. In addition, a recent study revealed that cytokines and growth factors produced by microglia regulate the development of ODCs (Beck et al., 1995; Lloyd and Miron, 2019). We observed a very moderate change in the levels of some cytokines. It is conceivable that suppression of autophagy may alter the basal level of expression of cytokines and growth factors in microglia, which in turn could upregulate the development of ODCs. This will be a focus of future studies.

Apart from altered homeostasis of ODCs, we also observed altered size of the nodes of Ranvier in ATG7KO mice. This may reflect the change in myelination. Indeed, loss of TSC1 in myelinating cells was reported to cause downregulation of quaking and neurofascin, leading to altered nodes of Ranvier along the axons (Pillai et al., 2009; Shi et al., 2018). The nodes of Ranvier play a critical role in propagation of action potential along the axon. It is of great interest to elucidate how impaired microglial autophagy leads to altered size of the nodes of Ranvier.

### Myelination in seizure propagation

Elevated levels of mature ODCs and myelin proteins and increased seizure susceptibility are two overt phenotypic changes in ATG7KO mice. Although it is nearly impossible to prove that the altered myelination is the underlying cause of increased seizure susceptibility, there is a growing evidence to suggest that altered myelination constitutes part of the mechanism underlying epilepsy. Hypomyelination was observed in TSC1 and epileptic conditions (Meikle et al., 2007; Shepherd et al., 2013; Carson et al., 2015; Ercan et al., 2017; Scholl et al., 2017; Schurr et al., 2017; Larson et al., 2018; Sharma et al., 2018; Peters et al., 2019). Loss of function of genes in ODCs was sufficient to cause spontaneous seizures (Lebrun-Julien et al., 2014; Wahl et al., 2014; Figlia et al., 2017; McLane et al., 2017; Shi et al., 2018). Myelination plays a considerable role in determining the rate of stationary transmission of action potentials (Arancibia-Cárcamo et al., 2017). Changes in myelination modify brain networks and promote the propagation of seizures (Larson et al., 2018). White matter structures are heavily myelinated and have long been known to be involved in propagation of epileptiform activity between various areas of the cerebral cortices (McCaughran et al., 1976, 1978). We found that autophagy-deficient animals display increased numbers of mature ODCs and higher levels of myelin proteins, and develop severe seizures. Our findings may explain why seizure activities are readily spread to the contralateral side of the brain and why autophagy-deficient animals are prone to developing fatal generalized seizures.

Apart from the forebrain commissures, which have been implicated in epileptogenesis and seizure propagation, additional evidence suggests that extracommissural routes, i.e., limbic–brainstem connections, may serve as an alternative path for the spreading and development of severe tonic–clonic seizures and epilepsy-associated mortality (Wada and Sato, 1975). As brainstem seizures have a profound impact on mortality, it is conceivable that myelination in these commissural and extracommissural connections could be changed, leading to facilitated propagation of seizures into the brainstem and resulting in the generalized tonic–clonic seizures and very high mortality seen in autophagy-deficient mice. Future studies will characterize myelination in these structures.

Of particular note, despite the stimulation-evoked large-amplitude electrical activities detected in the ipsilateral cortex in both control and ATG7KO mice, we did not see any epileptiform activities in the ipsilateral cortex. These data suggest that the ipsilateral cortex is inhibited on amygdala stimulation, likely because of ipsilateral cortical spreading of depression (Kelly et al., 1999), and the seizures only readily propagate to the contralateral cortex. Interestingly, mice developed generalized behavioral seizures even in the absence of ipsilateral seizure activity. This suggests that generalized behavioral seizures can be independent of cortical activation, indicating potential involvement of subcortical and brainstem structures in generalized seizures.

A growing body of evidence demonstrates that microglia influence neuronal excitability and seizure susceptibility (Badimon et al., 2020; Umpierre et al., 2020; Wu et al., 2020). We found that excessive activation of mTOR in TSC1-deficient mice leads to severe spontaneous seizures, involving a noninflammatory mechanism (Zhao et al., 2018). Our recent study revealed that deletion of microglial mTOR increases excitatory neuronal death and spontaneous recurrent seizures (Zhao et al., 2020). Here, we found that deletion of Atg7 in microglia promotes increased amounts of mature ODCs and myelination markers. Interestingly, hypomyelination was reported in both human TSC1 patients and TSC1-deficient animal models (Meikle et al., 2007; Shepherd et al., 2013; Carson et al., 2015; Ercan et al., 2017; Scholl et al., 2017; Schurr et al., 2017; Larson et al., 2018; Sharma et al., 2018; Peters et al., 2019). Because mTOR signaling and autophagy are connected, the contrasting outcomes are intriguing and perhaps reflect the different cell types in which the mTOR or autophagy signaling is altered. For example, in a previous study in a mouse model, TSC1 was deleted in neurons (Meikle et al., 2007). Also, in human TSC1 patients, TSC1 is presumably mutated in neurons (and all other cells), resulting in large balloon-like cells. In contrast, in the present study, ATG7 was restrictively deleted in microglia. It is conceivable that mTOR and autophagy signaling in neurons has opposite effects on ODC homeostasis compared with signaling in microglia.

Apart from impacting myelination and ODCs, we acknowledge that microglial autophagy deficiency could also impact CNS homeostasis through other means, leading to increased seizure susceptibility and high mortality. For example, inactivation of microglial autophagy was reported to impair synaptic pruning and learning behavior (Kim et al., 2017). Thus, autophagy deficiency may alter synaptic pruning of microglia, which could in turn impact neuronal circuitry development and maturation, resulting in increased seizure susceptibility. Moreover, although we did not see a significant change in the density of astrocytes, astrocytes still could be impacted by autophagy deficiency in microglia and the homeostatic activity of astrocytes could be compromised, resulting in severe seizures. Finally, we observed a moderate increase of cytokines in ATG7KO mice. Cytokines have been implicated in seizures (Vezzani et al., 2011). It is conceivable that elevated cytokines may contribute to the increased seizure severity in ATG7KO mice.

The Cx3cr1-cre line mediates the deletion of ATG7KO in microglia during early stages of brain development, which likely has a broad impact on the CNS. Therefore, an ideal approach would be to employ an inducible Cx3cr1-creERT line to delete ATG7KO in the mature brain. However, our recent study revealed that the Cx3cr1-CreER inducible line has spontaneous leakage (Zhao et al., 2019). We found that up to 25% of microglia express GFP reporter before tamoxifen treatment. This prohibits us from pursuing this approach. In the present study, the impact of microglial deletion of ATG7 on myelination and ODCs was characterized in the mature brain. Previous studies reported that microglia regulate ODC proliferation and myelination of neuronal axons in early brain development (Miron, 2017; Wlodarczyk et al., 2017; Lloyd and Miron, 2019). Future studies will examine whether microglial ATG7KO deletion has any impact on myelination and the density of ODCs in the developing brain.

Cx3Cr1 is also expressed in some peripheral monocytes, i.e., macrophages, including those of the perivasculature, choroid plexus and meninges. We recently reported an important role of autophagy in Cx3cr1+ mononuclear cells in intestinal fibrosis (Mathur et al., 2019). Thus, ATG7 is likely deleted in macrophages in ATG7KO (ATG7^Cx3Cr1-CreCKO^) mice. Although under non-perturbed conditions there is very little, if any, infiltration of macrophages into the brain, we cannot rule out the possibility that ATG7 deletion in macrophages impacts seizure susceptibility to convulsants.

In summary, microglial autophagy plays a critical role in maintaining CNS homeostasis. Impairment of microglial autophagy in ATG7KO mice results in severe generalized seizures leading to death. Histologically, the phenotype includes a significant increase of ODC density and myelin proteins in white matter regions of the brain. Our findings have significant implications in understanding the basis of, and developing treatments for, severe generalized and refractory seizures as well as epilepsy-associated mortality.

## Synthesis

Reviewing Editor: William Stacey, University of Michigan Decisions are customarily a result of the Reviewing Editor and the peer reviewers coming together and discussing their recommendations until a consensus is reached. When revisions are invited, a fact-based synthesis statement explaining their decision and outlining what is needed to prepare a revision will be listed below. Note: If this manuscript was transferred from JNeurosci and a decision was made to accept the manuscript without peer review, a brief statement to this effect will instead be what is listed below.

The reviewers and I have had a consultation and agree that the work is interesting and timely. We would like to have the following items addressed in a resubmission. Most of these are discussion items. Regarding #9, eNeuro tries to avoid asking for new experiments. The data would be very helpful to have in the paper if they are available, but you should not feel obliged to add it if the data are not available.

1. The Cx3cr1-cre line used in the study has a caveat for microglia specific study, as the mice express Cre constitutively and may affect peripheral macrophages. Thus, the behavioral test results could result from non-microglia effects. The authors should take a caution in interpreting the results and others. This notion can be included in the discussion.

2. The lack of the change of cell density and robust activation of microglia in Atg7Cx3Cr1-Cre KO mice are supported by previous reports (e.g. Choi, I et al, Nature Communications, 2020, PMID: 32170061). The authors can cite the reference and strengthen their observations.

3. A recent study by Berglund (Science Immunology, 2020, PMID: 33067381) has also described a role for microglial Atg7 gene in degrading myelin, which provides potential mechanistic insight into the current study. This report can be also cited.

4. The authors used a constitutive CX3CR1-cre used rather than a tamoxifen induced cre for conditional deletion of ATG in microglia. There was still a debate on the specificity of this constitutive cre line. This limitation should be discussed. Also, further work should consider the inducible model.

5. It would seem that the observed differences in myelination and oligodendrocytes occur during development. This should be discussed more in depth.

6. What are the potential mechanisms linking microglial activity to myelination and oligodendrocyte density? This should be discussed, especially in light of #1-3

7. The author claimed that the induction of SE required less pilocarpine in KO mice than the control mice. The results are important and should be shown. Please show pilocarpine dose data.

8. Even though the authors state that there was only a “moderate” increase in cytokine expression by ATG7 KO microglia, there is a significate body of literature that indicates that increased inflammation in the brain can influence seizure thresholds. This should be at least discussed in the manuscript.

9. It would also be of interest to determine how cytokine levels compare after kindling. If there is a way to include these data it would be very helpful. However, if the experiments are not available or not easily added, they are not a requirement for resubmission.

10. The authors should discuss the recent progress in studying microglia in seizures (PMID: 32621847; 32716294; 32999463) and how these are connected with microglial autophagy.
